# Real-world health outcomes in adults with moderate-to-severe psoriasis in the United States: a population study using electronic health records to examine patient-perceived treatment effectiveness, medication use, and healthcare resource utilization

**DOI:** 10.1186/s12895-018-0072-2

**Published:** 2018-06-28

**Authors:** April W. Armstrong, Shonda A. Foster, Brian S. Comer, Chen-Yen Lin, William Malatestinic, Russel Burge, Orin Goldblum

**Affiliations:** 1 Department of Dermatology, Keck School of Medicine, University of Southern California, Los Angeles, CA 90033 USA; 2 Eli Lilly and Company, Indianapolis, IN USA; 3 College of Pharmacy, University of Cincinnati, Cincinnati, OH USA

**Keywords:** Electronic health records, EHR, Psoriasis, Dermatology, Treatment effectiveness, Outcomes, Switching, Discontinuation, Healthcare resource utilization, Costs

## Abstract

**Background:**

Little is known regarding real-world health outcomes data among US psoriasis patients, but electronic health records (EHR) that collect structured data at point-of-care may provide opportunities to investigate real-world health outcomes among psoriasis patients. Our objective was to investigate patient-perceived treatment effectiveness, patterns of medication use (duration, switching, and/or discontinuation), healthcare resource utilization, and medication costs using real-world data from psoriasis patients.

**Methods:**

Data for adults (≥18-years) with a dermatology provider-given diagnosis of psoriasis from 9/2014–9/2015 were obtained from dermatology practices using a widely used US dermatology-specific EHR containing over 500,000 psoriasis patients. Disease severity was captured by static physician’s global assessment and body surface area. Patient-perceived treatment effectiveness was assessed by a pre-defined question. Treatment switching and duration were documented. Reasons for discontinuations were assessed using pre-defined selections. Healthcare resource utilization was defined by visit frequency and complexity.

**Results:**

From 82,621 patients with psoriasis during the study period, patient-perceived treatment effectiveness was investigated in 2200 patients. The proportion of patients reporting “strongly agree” when asked if their treatment was effective was highest for biologics (73%) and those reporting treatment adherence (55%). In 16,000 patients who received oral systemics and 21,087 patients who received biologics, median treatment duration was longer for those who received biologics (160 vs. 113 days, respectively). Treatment switching was less frequent among patients on systemic monotherapies compared to those on combination therapies. The most common reason for discontinuing biologics was loss of efficacy; the most common reason for discontinuing orals was side effects. In 28,754 patients, higher disease severity was associated with increased healthcare resource utilization (increased visit frequency and complexity). When compared between treatment groups (*n* = 10,454), healthcare resource utilization was highest for phototherapy. Annual medication costs were higher for biologics ($21,977) than oral systemics ($3413).

**Conclusions:**

Real-world research using a widely implemented dermatology EHR provided valuable insights on patient perceived treatment effectiveness, patterns of medication usage, healthcare resource utilization, and medication costs for psoriasis patients in the US. This study and others utilizing EHRs for real-world research may assist clinical and payer decisions regarding the management of psoriasis.

**Electronic supplementary material:**

The online version of this article (10.1186/s12895-018-0072-2) contains supplementary material, which is available to authorized users.

## Background

Psoriasis is an immune-mediated disease that affects 3.2% of adults in the United States (US) [1, 2]. The estimated percentages of patients in the US with mild, moderate, or severe psoriasis are 83.3, 11.4, and 5.3%, respectively [3]. Psoriasis is associated with significant morbidity and clinically significant comorbidities including diabetes, cardiovascular disease, metabolic syndrome, autoimmune diseases, and psychiatric impairment [4–10]. The disease impacts overall quality of life and productivity due to its physical and psychological components [11]. Currently, retrospective research in psoriasis is limited by knowledge gaps that exist in commonly used data sources, but electronic health records (EHR) may help address this problem [12].

Population studies in psoriasis have typically relied on claims databases or publicly available national databases [12]. However, these databases often lack point-of-care data collected at actual clinic visits. Furthermore, these databases are often not specialty-specific and therefore do not ask clinically relevant questions to a particular specialty [12]. Disease registries or post-marketing drug registries collect useful and structured information [13–16], but these registries may have circumscribed focus, assess a characteristically or geographically limited population, and/or require substantial human resources and financial support [12]. Specialty-specific EHRs provide an opportunity to conduct research from a large, diverse population using clinically relevant data that are collected at point-of-care as part of a typical provider consultation. Because specialty-specific EHRs are completed by specialist providers, the specialty-specific data are much less likely to be subject to misclassification errors compared to data collected by non-specialist providers. EHR systems that collect large amounts of structured data from diverse dermatology practices can fill substantial knowledge gaps not filled with claims databases, publicly available national databases, or data from single institutions [12].

In this study, we used point-of-care, real-world clinical data from a widely used dermatology-specific EHR in the US to examine patient-perceived treatment effectiveness, patterns of medication use (duration, switching, and/or discontinuation), healthcare-resource utilization, and medication costs among psoriasis patients.

## Methods

### Data source

Data were collected from Electronic Medical Assistant (EMA) Dermatology, a HIPAA-compliant dermatology-specific cloud-based EHR (Modernizing Medicine, Inc., Boca Raton, Florida, US). EMA is a widely implemented dermatology-specific EHR platform, used by over 4500 dermatology providers (30% of the market share) across the US. EMA houses data for over 500,000 psoriasis patients from 49 US states and 2 territories. Dermatology providers input data directly into this EHR during clinical visits at point-of-care. Data were de-identified to ensure patient privacy. Research using de-identified records was approved by the New England Independent Review Board.

### Study design

This multicentre, longitudinal, observational cohort study retrospectively examined adults (≥ 18 years) with psoriasis who visited participating dermatology practices in the US during the study period (September 1, 2014-September 1, 2015).

### Study population

The study population included adults who were diagnosed with psoriasis by a dermatologist, were classified as having moderate-to-severe psoriasis, and visited a dermatology provider during the study period. Patients were considered to have “moderate-to-severe” disease if they were scored ≥3 on the static physician’s global assessment (sPGA [0 = very clear, 5 = very severe]), ≥ 3% body surface area (BSA), received phototherapy, oral systemics (methotrexate, acitretin, cyclosporine, or apremilast), or biologic therapies (etanercept, adalimumab, infliximab, ustekinumab, or secukinumab) during the study period or 6 months prior to study initiation. Patients were categorized into the following treatment groups for analyses: topical treatments, phototherapy, oral systemic treatments (methotrexate, acitretin, cyclosporine, and apremilast), biologic treatments (i.e., etanercept, adalimumab, infliximab, ustekinumab, and secukinumab), combination treatments, and other interventions. Other interventions included patients who were not on topical, phototherapy, oral systemic, biologic, combination therapies commonly used for psoriasis, or patients not receiving psoriasis treatment during the study period. At the time of the study, limited data were available for secukinumab, and data were not available for ixekizumab or brodalumab.

### Patient-perceived treatment effectiveness

Patient-perceived treatment effectiveness was assessed by patient response to the following question at follow-up: “I believe this treatment is effective in clearing my skin of psoriasis.” Responses were graded on a 5-point Likert scale (1 = “strongly agree,” 5 = “strongly disagree”). Adherence was determined by a “yes” or “no” patient response in EMA to the following question: “The treatment was followed as directed.”

#### Patterns of medication usage: duration, switching, and discontinuation

##### Medication duration

Median duration was defined as the amount of time that a patient is on a study drug of interest. Medication duration was calculated for biologics (etanercept, adalimumab, and ustekinumab) and oral systemic medications (methotrexate, apremilast, acitretin, and cyclosporine) during the study period. Patients who received biologic (etanercept, adalimumab, and ustekinumab) or oral systemic medications (methotrexate, apremilast, acitretin, and cyclosporine) at any time during the study period were included in the analysis.

##### Changing systemic medications

Starting treatment was defined as the earliest treatment that a patient was on during the study period. A single treatment switch was defined as any change in treatment group. Patients that switched more than once were classified as undergoing multiple switches. Time on treatment was defined as the overall time documented in EMA on a given treatment prior to the first switch during the study period.

##### Reasons for discontinuing treatment

Providers documented the reasons for treatment discontinuation after conversations with the patient. The providers selected from “loss of efficacy,” “side effects,” “inability to comply with treatment regimen,” “inability to afford treatment,” “patient fear or risk,” or “unknown.” Providers could select more than one reason.

#### Healthcare resource utilization

##### Visit frequency

Visit frequency was defined as the number of patient visits during the study period.

##### Visit complexity

Visit complexity was defined as a function of annual combined visit and procedure costs using evaluation and management (E/M) (Additional file 1: Table S1) and Current Procedural Terminology (CPT) codes. Separately, annual visit costs were also calculated using only E/M codes.

##### Medication costs

Medication costs were calculated as average medication costs per patient per year (using standard National Average Drug Acquisition Cost pricing). Patients who received biologic (etanercept, adalimumab, and ustekinumab) or oral systemic medications (methotrexate, apremilast, acitretin, and cyclosporine) during the study period were included in the analysis, and those receiving combination treatment were counted more than once. Treatments were excluded if pricing data were unavailable (i.e. secukinumab, infliximab).

##### Data analyses

Data are presented as numbers and percentages, mean ± standard deviation (SD), or median (interquartile range [IQR]), where appropriate. All analyses were performed using R (version 3.2.2) [17]. To be included in the patient-perceived treatment effectiveness analyses, patients had to be on a given oral or biologic treatment for at least 6 months, except for cyclosporine for which patients were included regardless of treatment duration because cyclosporine is often used intermittently. Patient-perceived overall treatment effectiveness was evaluated with stratification by treatment group first and then further stratification by adherence. Only the most recent treatment satisfaction responses were used for patient-perceived treatment effectiveness analyses. Visit frequency and complexity were assessed in relation to maximum sPGA, maximum BSA, or treatment documented during the study period. Treatment group comparisons for visit frequency and costs only included patients that did not switch treatments during the study period.

## Results

### Patient-perceived treatment effectiveness

From 82,621 adult patients with psoriasis during the study period, patient-perceived treatment effectiveness was investigated in 2200 patients with psoriasis. Patient perceived treatment effectiveness response choices for the question “I believe this treatment is effective in clearing my skin of psoriasis” included “strongly agree,” “somewhat agree,” “neither agree or disagree,” “somewhat disagree,” and “strongly disagree.” Demographics and clinical characteristics for this patient population are listed in Additional file 1: Table S2. Mean age, comorbidities, and race were similar across the categories of patient-perceived treatment effectiveness. Overall 50% of patients strongly agreed that their treatment was effective compared to 32% reporting “somewhat agree” and 7% reporting “neither agree nor disagree” (Table 1). Biologics users reported highest agreement with the statement their treatment was effective (73%), followed by phototherapy (61%), and then oral systemics (57%).

**Table 1:** Patient-perceived treatment effectiveness stratified according to treatment groups

	Total (n)	Strongly Agree n (%)	Somewhat Agree n (%)	Neither Agree nor Disagree n (%)	Somewhat Disagree n (%)	Strongly Disagree n (%)
Total	2200	1099 (50.0)	706 (32.1)	161 (7.3)	150 (6.8)	84 (3.8)
Topical	1336	524 (39.2)	492 (36.8)	128 (9.6)	126 (9.4)	66 (4.9)
Phototherapy	175	106 (60.6)	63 (36.0)	†	†	†
Oral systemics	199	113 (56.8)	50 (25.1)	18 (9.1)	†	†
Biologics	478	350 (73.2)	96 (20.1)	†	12 (2.5)	†

n, number of patients; †Patient counts < 5 hidden to comply with HIPAA privacy rule. Additional cells hidden as needed to prevent recalculation of hidden values

Results for patient-perceived treatment effectiveness were also examined following stratification by treatment adherence (Table 2). Among patients who provided information on treatment effectiveness, 53% reported treatment adherence, 3% reported non-adherence, and 44% had no adherence data. Patients who were reported to be adherent to their treatments were more likely to perceive their treatments to be effective (84% in the adherent group compared to 62% in the non-adherent group). Similarly, those who were reported to be non-adherent to their treatment were more likely to perceive their treatment to be ineffective (23%) compared to the adherent patients (10%).

**Table 2:** Patient-perceived treatment effectiveness overall and stratified according to treatment group and treatment adherence

	Adherence		Strongly Agree	Somewhat Agree	Neither Agree nor Disagree	Somewhat Disagree	Strongly Disagree
Treatment		n (%)	n (%)	n (%)	n (%)	n (%)	n (%)
Total	Yes	1176 (53.5)	608 (51.7)	375 (31.9)	76 (6.5)	74 (6.3)	43 (3.7)
	No	66 (3.0)	12 (18.2)	29 (43.9)	10 (15.2)	7 (10.6)	8 (12.1)
	Unknown	958 (43.5)	479 (50.0)	302 (31.5)	75 (7.8)	69 (7.2)	33 (3.4)
Topical	Yes	738 (55.2)	306 (41.5)	267 (36.2)	67 (9.1)	63 (8.5)	35 (4.7)
	No	58 (4.3)	9 (15.5)	27 (46.6)	8 (13.8)	6 (10.3)	8 (13.8)
	Unknown	540 (40.4)	209 (38.7)	198 (36.7)	53 (9.8)	57 (10.6)	23 (4.3)
Phototherapy	Yes	88 (50.3)	54 (61.4)	†	†	†	†
	No	†	†	†	†	†	†
	Unknown	87 (49.7)	52 (59.8)	†	†	†	†
Oral Systemics	Yes	90 (45.2)	54 (60.0)	†	†	†	†
	No	†	†	†	†	†	†
	Unknown	106 (53.3)	58 (54.7)	†	13 (12.3)	†	†
Biologics	Yes	254 (53.1)	191 (75.2)	52 (20.5)	†	5 (2.0)	†
	No	5 (1.0)	†	†	†	†	†
	Unknown	219 (45.8)	157 (71.7)	43 (19.6)	8 (3.7)	6 (2.7)	5 (2.3)

*n* number of patients

^†^Patient counts < 5 hidden to comply with HIPAA privacy rule. Additional cells hidden as needed to prevent recalculation of hidden values

#### Patterns of medication usage: duration, switching, and discontinuation

We evaluated medication duration in 21,087 patients on biologics and 16,000 patients on oral systemics. Overall, patients on biologics had longer median duration on treatment compared to patients on oral systemic medications (160 days [IQR, 57–279] vs. 113 days [IQR, 50–217]; biologics vs orals, respectively).

We also examined medication use patterns for patients who started on monotherapies or combination therapies (Table 3). Overall, patients who started on monotherapies were least likely to switch with 58% of patients treated with topicals, 37% of patients treated with biologics, and 33% of patients treated with oral systemics reporting no treatment switch. Median treatment duration for patients that did not switch therapies was 327 days for topicals, 238 days for biologics, and 159 days for oral systemics. Switching multiple times during the study period was most common among patients who were on combination therapies. For example, patients treated with oral systemic + biologic + phototherapy, biologic + phototherapy, and oral systemic + phototherapy combinations experienced multiple treatment switches (100, 75, and 68%, respectively).Among 427 patients who reported discontinuing treatment, no major differences were observed for age, gender, or comorbidity with respect to treatment groups (Additional file 1: Table S3). Discontinuations were highest from biologic treatment (52%), followed by oral systemic treatment (34%), and phototherapy (6%), with 9% of patients reporting multiple discontinuations. Overall, the most common reasons reported for discontinuing treatment were loss of efficacy (60%) and side effects (27%) (Table 4). When discontinuation reasons were stratified by treatment, the most common reasons for discontinuing treatment were loss of efficacy for biologics (74%) and phototherapy (55%), and side effects for oral systemics (48%).

**Table 3:** Changes in treatment group from starting treatment for patients treated with monotherapy or combination therapy

Starting treatment group^a^	New treatment group	Percentage switched n (%)	Median (days)^b^	IQR
Topical, *n* = 44,603	Phototherapy	1613 (3.6)	48.0	0.0–296.0
	Oral Systemic	2232 (5.0)	139.0	14.0–327.3
	Biologic	2841 (6.4)	135.0	15.0–283.0
	Oral Systemic + Biologic	9 (0.0)	30.0	0.0–48.0
	Multiple Switch	11,927 (26.7)	79.0	7.0–243.0
	No Switch	25,981 (58.2)	327.0	174.8–365.0
Phototherapy, *n* = 5248	Topical	2438 (46.5)	84.0	33.0–185.0
	Oral Systemic	19 (0.4)	113.0	53.0–149.0
	Biologic	18 (0.3)	79.0	44.3–142.5
	Oral Systemic + Phototherapy	45 (0.9)	56.0	5.0–239.0
	Biologic + Phototherapy	17 (0.3)	76.0	21.0–189.0
	Other Interventions	665 (12.7)	35.0	3.0–97.0
	Multiple Switch	726 (13.8)	98.0	45.0–182.0
	No Switch	1320 (25.2)	160.0	55.0–350.7
Oral Systemic, *n* = 7116	Topical	1751 (24.6)	105.0	21.0–219.0
	Oral Systemic	7 (0.1)	0.0	0.0–111.0
	Biologic	6 (0.1)	34.5	7.0–134.8
	Oral Systemic + Phototherapy	19 (0.3)	2.0	0.0–166.5
	Oral Systemic + Biologic	73 (1.0)	35.0	0.0–100.0
	Other Interventions	445 (6.3)	33.0	0.0–136.0
	Multiple Switch	2459 (34.6)	117.0	45.0–215.5
	No Switch	2356 (33.1)	159.0	61.0–299.1
Biologic, *n* = 11,767	Topical	1857 (15.8)	191.0	83.0–290.2
	Oral Systemic	5 (0.0)	317.0	0.0–342.2
	Biologic	5 (0.0)	42.0	39.0–87.0
	Biologic + Phototherapy	15 (0.1)	7.0	0.0–204.9
	Oral Systemic + Biologic	80 (0.7)	204.8	39.5–302.5
	Other Interventions	800 (6.8)	0.0	0.0–186.6
	Multiple Switch	4669 (39.7)	112.0	15.0–227.0
	No Switch	4336 (36.9)	238.0	139.0–319.8
Oral Systemic + Phototherapy, *n* = 146	Topical	10 (6.9)	52.5	36.5–141.5
	Phototherapy	10 (6.9)	165.0	146.8–240.6
	Oral Systemic	14 (9.6)	88.5	31.0–132.8
	Multiple Switch	99 (67.8)	90.0	40.5–156.0
	No Switch	13 (8.9)	54.0	20.0–144.0
Biologic + Phototherapy, *n* = 65	Biologic	9 (12.0)	91.0	28.0–227.0
	Multiple Switch	56 (74.7)	70.0	38.8–148.0
Oral Systemic + Biologic, *n* = 350	Oral Systemic	18 (5.1)	81.5	3.8–210.5
	Biologic	54 (15.2)	144.5	49.8–227.0
	Multiple Switch	237 (66.8)	94.0	35.0–189.0
	No Switch	41 (11.6)	140.1	48.0–298.5
Oral Systemic + Biologic + Phototherapy, *n* = 5	Multiple Switch	5 (100.0)	137.0	109.0–196.0
Other Interventions, *n* = 13,200	Topical	1782 (13.5)	25.5	0.0–140.0
	Phototherapy	408 (3.1)	0.0	0.0–0.0
	Oral Systemic	491 (3.7)	0.0	0.0–86.0
	Biologic	1488 (11.3)	12.0	0.0–158.3
	Multiple Switch	4835 (36.6)	13.0	0.0–102.5
	No Switch	4196 (31.8)	265.0	115.9–365.0

*IQR* interquartile range

^a^Patient numbers provided for starting treatment as documented in EMA for the study period

^b^For patients tracked in EMA prior to the study initiation date, their first visit date was captured and time on treatment was normalized to account for overall time documented in EMA

**Table 4:** Reasons for discontinuing treatments (*n* = 528)

Treatment	Patient/Fear Risk	Inability to afford treatment	Inability to comply	Loss of efficacy	Side effects
	n (%)	n (%)	n (%)	n (%)	n (%)
Total	30 (5.7)	28 (5.3)	13 (2.5)	314 (59.5)	143 (27.1)
Phototherapy	†	†	†	18 (54.5)	8 (24.2)
Oral systemics	†	†	†	81 (39.9)	98 (48.3)
Biologics	17 (5.8)	16 (5.5)	7 (2.4)	215 (73.6)	37 (12.7)

*n* number of patients

^†^Patient counts hidden to comply with the HIPAA privacy rule. Additional cells hidden as needed to prevent recalculation of hidden values

#### Healthcare resource utilization

##### Visit frequency

**Fig. 1:**
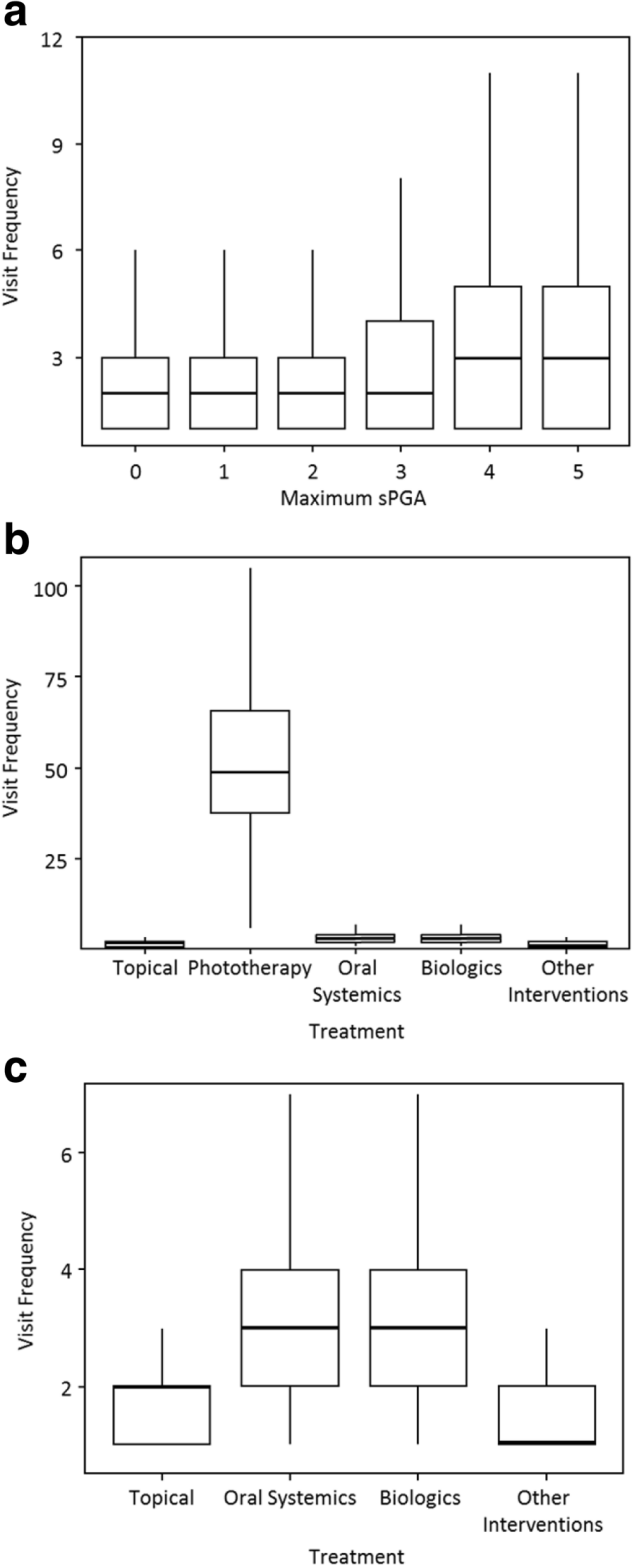
Visit frequency stratified according to maximum sPGA (**a**), treatment (**b**), and treatment with phototherapy excluded (**c**)

Visit frequency is a measure of healthcare resource utilization. We evaluated visit frequency in patients with data available for maximum disease severity and/or treatment. Among 28,754 patients who had sPGA data, those with severe psoriasis (sPGA of 4 or 5) had a greater frequency of visits compared to those with mild-to-moderate psoriasis (sPGA of 0–3) (Fig. 1a and Additional file 1: Table S4). Among 27,150 patients who had BSA data, the distribution of the visit frequency data reflected a trend consistent with the sPGA data (Results not shown).

Visit frequency during the study period was also stratified for 10,454 patients based on treatment groups (Fig. 1c/d and Additional file 1: Table S5). Patients receiving phototherapy had the highest median number of visits during the one- year study period (49). Biologics and oral systemic treatments both had a median of 3 visits over the 12-months study period. Patients receiving topical treatments alone or other interventions had the lowest median visit frequencies, 2 and 1, respectively.

##### Visit complexity

Visit complexity was defined by patient annual healthcare costs using both visit and procedure costs. This definition was based upon the assumption that patients with greater healthcare costs have greater visit complexity. As with visit frequency, we evaluated visit complexity in patients with data available for maximum disease severity and/or treatment. Patients with a severe psoriasis (sPGA of 4 or 5) had the highest annual combined costs of visits and procedures with $161.70 and $150.92, respectively (Table 5). The distribution of the data in patients with BSA data available reflected a trend consistent with the sPGA data (Results not shown). Similar results were observed for patients with data available for sPGA (Additional file 1: Table S6) and BSA (Results not shown) when comparing disease severity to only annual visit costs.

**Table 5:** Annual combined visit and procedure costs stratified according to maximum sPGA

	Minimum ($)	Maximum ($)	Median ($)	IQR ($)
Maximum sPGA				
0	20.12	892.56	88.40	73.30–109.60
1	20.12	5633.94	104.93	73.30–146.60
2	20.12	10,317.27	108.88	73.30–182.18
3	20.12	15,619.27	109.60	75.46–219.20
4	20.12	17,735.86	161.70	108.88–293.20
5	7.91	9353.18	150.92	108.88–300.94

*sPGA* static physicians global assessment, *IQR* interquartile range

Annual combined costs of visits and procedures were also compared between the different treatment groups (Table 6). Patients who received phototherapy had the highest median annual combined visit and procedure costs ($3217.98). Patients who received other interventions had the lowest median annual combined visit and procedure costs ($108.88). A comparison of treatment groups by only annual visit costs indicated that patients who received oral systemics had the highest median annual visit costs ($217.76) while patients receiving topicals or other interventions had the lowest median annual visit costs ($108.88 for both) (Additional file 1: Table S7).

**Table 6:** Annual combined visit and procedure costs stratified according to treatment groups

Treatment	Minimum ($)	Maximum ($)	Median ($)	IQR ($)
Topical	7.91	1999.68	117.50	73.30–214.16
Phototherapy	144.46	15,119.08	3217.98	1456.65–5678.06
Oral Systemics	25.51	1821.80	217.76	146.60–293.20
Biologics	25.51	1128.32	168.52	108.88–246.22
Other Interventions	20.12	936.07	108.88	73.30–182.18

*IQR* interquartile range

##### Medication cost

Medication costs were investigated for patients on oral systemic medications (*n* = 16,000) and those on biologic medications (*n* = 21,087). Patients on biologics had numerically higher average drug costs per patient relative to patients on oral systemics ($21,976.6 vs. $3412.71, respectively).

## Discussion

Health outcomes of psoriasis patients in the real world are critical to inform clinical practice. However, these data are scarce in the US. This is due at least in part to difficulties in synthesizing data across disparate EHR systems. Studies using structured, point-of-care clinical data that are supplied directly by dermatology providers can provide in-depth understanding of clinical interactions occurring at the visit-level in the real world.

This study uses real-world, point-of-care data obtained from dermatology providers on a widely used dermatology specific EHR-platform in the US in order to address clinically relevant questions in patients with moderate-to-severe psoriasis. Many data elements are unique to the present study, such as patient-perceived treatment effectiveness and collection of validated psoriasis outcome measures.

Patient-perceived treatment effectiveness affects clinical decision-making in substantial ways because patient input during clinical encounters often influence management plans. In this study, the majority of patients under the care of dermatology providers agreed that their treatments were effective. Specifically, those receiving biologic medications reported the highest rate of strong agreement that the treatment “is effective in clearing my skin of psoriasis.” The results derived from EMA for patient-perceived treatment effectiveness on biologics are in agreement with published studies [18]. Of note, greater patient-perceived treatment effectiveness was associated with treatment adherence. Because perceived treatment effectiveness and treatment adherence are interdependent, addressing treatment adherence directly with patients during visits is paramount.

The finding from this study that the majority of psoriasis patients thought their treatment was effective differs somewhat from a previous study [19]. The previous study consisted of randomly selected psoriasis patients regardless of provider type; more than half of these patients reported dissatisfaction with their treatment [19]. Two factors may contribute to the differences in findings. First, it is likely that patients under the care of dermatology providers are more likely to be satisfied with their treatments than patients cared for by generalists. Second, temporal differences in the study periods can affect the findings because the availability of advanced therapies for psoriasis has improved over time.

We investigated changes in treatments including switching and discontinuation. The data showed that both treatment switching and discontinuation were common in the study population. Switching was lowest in patients treated with topical therapies even though the highest proportion of those on topical therapies reported that they did not perceive their topical treatment to be effective. One potential explanation is that, even though topical therapies may only be modestly effective, patients continue on them due to perception of these therapies being safer. This explanation is supported by results from Armstrong and colleagues [19] indicating that a high proportion of psoriasis patients participating in National Psoriasis Foundation (NPF) Surveys with all spectrum of disease severity chose to receive topical treatment alone due to “fewer adverse events than other treatments”. Another potential explanation is that certain providers may have a higher threshold for what may trigger a therapeutic escalation from topical medications alone to initiating systemic therapies.

Across treatment categories, the most frequent reasons reported for discontinuation of treatment were loss of efficacy and side effects. Those on biologics or oral systemics reported the highest frequencies for treatment discontinuation. Among patients who discontinued biologics and oral systemics, the most common reason for discontinuing biologics was loss of efficacy, and the most common reason for discontinuing oral medications was side effects. Similarly, Levin and colleagues [20] also observed that discontinuations were most frequently due to lack of efficacy for biologics and adverse events for traditional systemic therapies. These results may help providers better understand why patients continue, discontinue, or switch treatments. In addition, these results highlight the need for newer treatments (biologics and oral) that are more capable of providing long-term disease control.

With regards to healthcare resource utilization, patients with more severe disease had greater visit frequency and visit complexity compared to those with milder psoriasis. Therefore, concerted efforts at controlling psoriasis severity are important not only to reduce physical and psychosocial burden, but also to reduce overall healthcare resource utilization. The association between severity and visit complexity (measured as cost) is consistent with findings by Evans [21]. Evans reported that patients with moderate-to-severe psoriasis have 5-fold higher total healthcare costs versus patients with mild psoriasis [21].

In addition, healthcare resource utilization also varied across treatment groups. Notably, being on oral therapies required highest visit complexity, possibly due to the need for more intense monitoring and management, greater frequency of laboratory evaluation, and greater incidence of adverse events [22]. While treatment with biologics was associated with higher costs, biologic medication duration was longer than oral systemics. Phototherapy was associated with the highest visit frequency, which was expected due to the regularity of visits necessary for in-office light treatments. These results highlight the differences in healthcare resource utilization and costs associated with differing levels of disease severity and treatments.

This pilot study highlights the potential for using an EHR such as EMA to conduct real-world health outcomes research. A unique advantage of a dermatology-specific EHR is the minimization of the risk of misclassification because the data are entered directly by dermatology providers. This is supported by a prior study which reported psoriasis diagnoses (as ≥3 ICD-9 codes) provided by dermatologists had a 97.7% positive prediction value [23]. Both combination therapy data and patient adherence data were limited. In addition, documented medications reflect prescription behaviours and may not accurately represent ultimate utilization by patients. Our grouping of apremilast with other oral systemics may affect cost and healthcare utilization data.

## Conclusions

This study used point-of-care, real-world data from a widely implemented US EHR platform to examine several clinically relevant and important questions including patient-perceived treatment effectiveness, medication duration, reasons for switching/discontinuation of treatments, healthcare resource utilization, and costs. With continued development and improvement, EHRs with structured and validated data can serve as a powerful tool for real-world research. Real-world research using EHRs provides valuable insights and help clinicians and payers address questions on treatment patterns, costs of care, real-world effectiveness of treatments, and patient satisfaction in clinical practice.

## Additional file

Additional file 1: **Table S1**. E/M Codes. **Table S2**. Patient perception of treatment effectiveness stratified according to demographic and patient characteristics. **Table S3**. Demographic and patient characteristics of patients who discontinued treatment. **Table S4**. Visit frequency during the study period (9/14-9/15) stratified according to maximum sPGA. **Table S5**. Visit frequency during the study period (9/14-9/15) stratified according to treatment group. **Table S6**. Annual visit costs stratified according to maximum sPGA. **Table S7**. Annual visit costs stratified according to treatment. (DOCX 23 kb)

## Abbreviations

BSA: Body surface area
CPT: Current procedural terminology
E/M: Evaluation and management
EHR: Electronic health records
EMA: Electronic Medical Assistant
FDA: Food and Drug Administration
HIPAA: Health Insurance Portability and Accountability Act
IQR: Interquartile range
n: Number of patients
SD: Standard deviation
sPGA: Static physician global assessment
