# Supplementary material for: Real-world health outcomes in adults with moderate-to-severe psoriasis in the United States: a population study using electronic health records to examine patient-perceived treatment effectiveness, medication use, and healthcare resource utilization
Source: BMC Dermatol. 2018 Jun 28;18:4. doi: 10.1186/s12895-018-0072-2 (PMC6025830; doi:10.1186/s12895-018-0072-2)
Supplement: Supplementary file 1 — Table S1. E/M Codes. Table S2. Patient perception of treatment effectiveness stratified according to demographic and patient characteristics. Table S3. Demographic and patient characteristics of patients who discontinued treatment. Table S4. Visit frequency during the study period (9/14-9/15) stratified according to maximum sPGA. Table S5. Visit frequency during the study period (9/14-9/15) stratified according to treatment group. Table S6. Annual visit costs stratified according to maximum sPGA. Table S7. Annual visit costs stratified according to treatment. (DOCX 23 kb) [file 12895_2018_72_MOESM1_ESM.docx]

Additional file 1

Table S1. E/M Codes

| **E/M code** | **Short description** | **Non-facility price** |
| --- | --- | --- |
| **99201** | Office/outpatient visit new | $44.20 |
| **99202** | Office/outpatient visit new | $75.46 |
| **99203** | Office/outpatient visit new | $109.60 |
| **99204** | Office/outpatient visit new | $166.73 |
| **99205** | Office/outpatient visit new | $209.49 |
| **99211** | Office/outpatient visit est | $20.12 |
| **99212** | Office/outpatient visit est | $44.20 |
| **99213** | Office/outpatient visit est | $73.30 |
| **99214** | Office/outpatient visit est | $108.88 |
| **99215** | Office/outpatient visit est | $146.97 |

Available at: <https://www.cms.gov/apps/physician-fee-schedule/> [retrieved Nov 30, 2015].

Est, established

Table S2. Patient perception of treatment effectiveness stratified according to demographic and patient characteristics.

| **Variable** |  | **Total** | **Strongly agree** | **Somewhat agree** | **Neither agree nor disagree** | **Somewhat disagree** | **Strongly disagree** |
| --- | --- | --- | --- | --- | --- | --- | --- |
| **Total, n** |  | 2200 | 1099 | 706 | 161 | 150 | 84 |
| **Age (years), mean (SD)** |  | 55.8 (15.7) | 55.5(15.1) | 56.2 (16) | 55.5 (16.6) | 57.2 (16.7) | 54.2 (16.7) |
| **Gender, n (%)** |  |  |  |  |  |  |  |
|  | Female | 1152 | 535 (48.7) | 391 (55.4) | 86 (53.4) | 91 (60.7) | 49 (58.3) |
|  | Male | 1048 | 564 (51.3) | 315 (44.6) | 75 (46.6) | 59 (39.3) | 35 (41.7) |
| **Race, n (%)** |  |  |  |  |  |  |  |
|  | White | 1465 | 735 (66.9) | 476 (67.4) | 102 (63.4) | 95 (63.3) | 57 (67.9) |
|  | African-American | 72 | 33 (3) | 20 (2.8) | † | † | † |
|  | Asian | 47 | 16 (1.5) | 19 (2.7) | † | † | † |
|  | Hispanic | 135 | 61 (5.6) | 49 (6.9) | 10 (6.2) | 7 (4.7) | 8 (9.5) |
|  | Other | 182 | 97 (8.8) | 52 (7.4) | 14 (8.7) | 13 (8.7) | 6 (7.1) |
|  | Unknown | 299 | 157 (14.3) | 90 (12.7) | 20 (12.4) | 23 (15.3) | 9 (10.7) |
| **Arthritis** |  | 577 | 277 (25.2) | 200 (28.3) | 37 (23) | 37 (24.7) | 26 (31.0) |
| **Diabetes** |  | 340 | 164 (14.9) | 118 (16.7) | 22 (13.7) | 20 (13.3) | 16 (19.0) |
| **Depression** |  | 247 | 112 (10.2) | 84 (11.9) | 17 (10.6) | 24 (16) | 10 (11.9) |
| **Cardiovascular disease** |  | 818 | 390 (35.5) | 294 (41.6) | 56 (34.8) | 49 (32.7) | 29 (34.5) |
| **Liver disease** |  | 31 | 11 (1) | 11 (1.6) | † | † | † |
| **Lymphoma** |  | 9 | † | † | † | † | † |

†Patient counts <5 hidden to comply with HIPAA privacy rule. Additional cells hidden as needed to prevent recalculation of hidden values.

Table S3. Demographic and patient characteristics of patients who discontinued treatment.

| **Variable** |  | **Total** | **Phototherapy** | **Oral systemic** | **Biologic** | **Multiple** |
| --- | --- | --- | --- | --- | --- | --- |
| **Totals, n (%)** |  | 427 | 26 (6.1) | 143 (33.5) | 221 (51.8) | 37 (8.7) |
| **Age (years), mean (SD)** |  | 52 (14.4) | 54.3 (16.7) | 54.1 (14.8) | 50.3 (13.6) | 52.6 (15.8) |
| **Gender, n (%)** |  |  |  |  |  |  |
|  | Female | 220 | 12 (46.2) | 70 (49.0) | 120 (54.3) | 18 (48.6) |
|  | Male | 207 | 14 (53.8) | 73 (51.0) | 101 (45.7) | 19 (51.4) |
| **Race, n (%)** |  |  |  |  |  |  |
|  | White | 278 | 17 (65.4) | 90 (62.9) | 146 (66.1) | 25 (67.6) |
|  | African-American | 16 | † | † | † | † |
|  | Asian | 15 | † | 8 (5.6) | † | † |
|  | Hispanic | 17 | † | 5 (3.5) | † | † |
|  | Other | 24 | † | † | 14 (6.3) | † |
|  | Unknown | 77 | † | 29 (20.3) | 36 (16.3) | † |
| **Arthritis, n (%)** |  | 123 | 7 (26.9) | 39 (27.3) | 66 (29.9) | 11 (29.7) |
| **Diabetes, n (%)** |  | 60 | 7 (26.9) | 18 (12.6) | 29 (13.1) | 6 (16.2) |
| **Depression, n (%)** |  | 52 | 5 (19.2) | 10 (7.0) | 32 (14.5) | 5 (13.5) |
| **Cardiovascular disease, n (%)** |  | 162 | 10 (38.5) | 48 (33.6) | 93 (42.1) | 11 (29.7) |
| **Liver disease, n (%)** |  | † | † | † | † | † |
| **Lymphoma, n (%)** |  | † | † | † | † | † |

†Patient counts <5 hidden to comply with HIPAA privacy rule. Additional cells hidden as needed to prevent recalculation of hidden values.

Table S4. Visit frequency during the study period (9/14-9/15) stratified according to maximum sPGA.

|  | **Maximum sPGA** | | | | | |
| --- | --- | --- | --- | --- | --- | --- |
|  | **0** | **1** | **2** | **3** | **4** | **5** |
| **Visit frequency** |  | | | | | |
| **Total** | 1521 | 4385 | 6601 | 12130 | 3086 | 1031 |
| **(0-1]** | 524 | 1639 | 2639 | 4687 | 871 | 267 |
| **(1-2]** | 424 | 1176 | 1634 | 2752 | 656 | 215 |
| **(2-3]** | 232 | 600 | 847 | 1585 | 442 | 156 |
| **(3-4]** | 190 | 462 | 580 | 989 | 336 | 126 |
| **(4-5]** | 71 | 156 | 241 | 561 | 220 | 75 |
| **(5-10]** | 42 | 127 | 272 | 659 | 280 | 112 |
| **(10-20]** | 18 | 63 | 134 | 363 | 115 | 33 |
| **(20-131]** | 20 | 162 | 254 | 534 | 166 | 47 |

sPGA, static physician’s global assessment

Table S5. Visit frequency during the study period (9/14-9/15) stratified according to treatment group.

|  | **Treatment group** | | | | |
| --- | --- | --- | --- | --- | --- |
|  | **Topical** | **Phototherapy** | **Oral systemic** | **Biologic** | **Other interventions** |
| **Visit frequency** |  |  |  |  |  |
| **Total** | 8680 | 135 | 204 | 759 | 676 |
| (0-1] | 4322 | ^†^ | 20 | 111 | 357 |
| (1-2] | 2377 | ^†^ | 70 | 198 | 181 |
| (2-3] | 1027 | ^†^ | 53 | 97 | 57 |
| (3-4] | 503 | ^†^ | 42 | 224 | 38 |
| (4-5] | 230 | ^†^ | 9 | 89 | ^†^ |
| (5-10] | 202 | ^†^ | ^†^ | ^†^ | 28 |
| (10-20] | ^†^ | ^†^ | ^†^ | ^†^ | ^†^ |
| (20-50] | ^†^ | 65 | ^†^ | ^†^ | ^†^ |
| (50-121] | ^†^ | 60 | ^†^ | ^†^ | ^†^ |

†Patient counts <5 hidden to comply with HIPAA privacy rule. Additional cells hidden as needed to prevent recalculation of hidden values.

Table S6. Annual visit costs stratified according to maximum sPGA

|  | **Minimum**  **($)** | **Maximum**  **($)** | **Median**  **($)** | **IQR**  **($)** |
| --- | --- | --- | --- | --- |
| **Maximum sPGA** |  |  |  |  |
| **0** | $20.12 | $653.28 | $108.88 | $73.30–109.60 |
| **1** | $20.12 | $2,399.64 | $108.88 | $73.30–146.60 |
| **2** | $20.12 | $2,077.36 | $108.88 | $73.30–182.18 |
| **3** | $20.12 | $3,353.28 | $109.60 | $75.46–217.76 |
| **4** | $20.12 | $2,327.08 | $148.76 | $108.88–256.20 |
| **5** | $20.12 | $2,331.36 | $146.60 | $108.88–291.06 |

sPGA, static physicians global assessment; IQR, interquartile range

Table S7. Annual visit costs stratified according to treatment

|  | **Minimum**  **($)** | **Maximum**  **($)** | **Median**  **($)** | **IQR**  **($)** |
| --- | --- | --- | --- | --- |
| **Treatment group** |  |  |  |  |
| **Topical** | $20.12 | $1948.66 | $108.88 | $73.30–182.18 |
| **Phototherapy** | $44.20 | $1521.94 | $146.60 | $88.40–291.06 |
| **Oral systemic** | $44.20 | $1821.8 | $217.76 | $146.60–293.20 |
| **Biologic** | $20.12 | $1088.8 | $166.72 | $108.88–219.90 |
| **Other interventions** | $20.12 | $733.06 | $108.88 | $73.30–176.80 |

IQR, interquartile range
